# Case Report of Patients with Acute Respiratory Distress Syndrome Caused by COVID-19: Successfully Treated by Venovenous Extracorporeal Membrane Oxygenation and an Ultra-Protective Ventilation

**DOI:** 10.3390/medicina56110570

**Published:** 2020-10-29

**Authors:** Mi Hwa Park, Ah Jin Kim, Man-Jong Lee, Young Sam Kim, Jung Soo Kim

**Affiliations:** 1 Division of Pulmonology, Department of Internal Medicine, Inha University Hospital, Incheon 22332, Korea; bami.park@gmail.com; 2 Division of Critical Care Medicine, Department of Internal Medicine, Inha University Hospital, Incheon 22332, Korea; emjin23@naver.com (A.J.K.); likeavirgin@daum.net (M.-J.L.); 3 Division of Thoracic Surgery, Inha University Hospital, Incheon 22332, Korea; kimyoungsam@inha.ac.kr

**Keywords:** COVID-19, acute respiratory distress syndrome, venovenous extracorporeal membrane oxygenation, ultra-protective ventilation

## Abstract

Coronavirus disease (COVID-19) started in Wuhan (China) at the end of 2019, and then increased rapidly. In patients with severe acute respiratory distress syndrome (ARDS) caused by COVID-19, venovenous extracorporeal membrane oxygenation (VV-ECMO) is considered a rescue therapy that provides adequate gas exchange. The way in which mechanical ventilation is applied during VV-ECMO is not clear, however it is associated with prognosis. Currently, the mortality rate of COVID-19 patients that receive VV-ECMO stands at approximately 50%. Here, we report three patients that successfully recovered from COVID-19-induced ARDS after VV-ECMO and implementation of an ultra-protective ventilation. This ventilation strategy involved maintaining a peak inspiratory pressure of ≤20 cmH_2_O and a positive end-expiratory pressure (PEEP) of ≤ 10 cmH_2_O, which are lower values than have been previously reported. Thus, we suggest that this ultra-protective ventilation be considered during VV-ECMO as it minimizes the ventilator-induced lung injury.

## 1. Introduction

After the outbreak of coronavirus disease (COVID-19) caused by severe acute respiratory syndrome coronavirus 2 (SARS-Cov2) infection in Wuhan (China) in December 2019, it spread worldwide, and as of 20 July 2020 had claimed more than 600,000 lives [1]. In COVID-19 acute respiratory distress syndrome (ARDS), Gattinoni and colleagues proposed a new phenotype “Type L”, features of respiratory compliance close to normal. In contrast, respiratory compliance is low, because of non-cardiogenic pulmonary edema, shunt-related hypoxemia, and reduced lung size in classical ARDS (“Type H”). Type L could progress to Type H [2]. The COVID-19 pandemic has resulted in massive increases in the number of patients with acute respiratory distress syndrome (ARDS) in seriously affected areas and presents a global challenge to intensivists and intensive care units (ICUs). About 16% of patients with SARS-Cov2-infected pneumonia are transferred to ICUs because of ARDS [3] and the adoption of mechanical ventilation (MV) and a lung-protective ventilation strategy is strongly recommended for such patients [4]. Furthermore, when MV cannot provide sufficient gas exchange, venovenous extracorporeal membrane oxygenation (VV-ECMO) might provide a rescue therapy [5]. Previous outbreaks of respiratory viral disease, such as severe acute respiratory syndrome, pandemic H1N1, and Middle East respiratory syndrome, have provided positive insights of VV-ECMO. However, MV strategies during VV-ECMO in respiratory failure patients with COVID-19 are not clear. Pham et al. reported that in 123 H1N1-induced ARDS patients, an ultra-protective MV with VV-ECMO minimized alveolar pressure and subsequent lung injury and were associated with better outcomes than conventional MV with VV-ECMO [6]. Here, we describe three patients of COVID-induced ARDS successfully treated with VV-ECMO and maintained using an ultra-protective MV until weaned off VV-ECMO.

## 2. Case Report

All three patients were diagnosed with COVID-19 infection by polymerase chain reaction (AllplexTM 2019-nCoV Assay^TM^; Seegene Co., Seoul, Korea).

After VV-ECMO application, pump flow was adjusted to a target percutaneous oxygen saturation of 95%. Sweep gas flow was titrated to a target arterial carbon dioxide partial pressure of 45 cmH_2_O, and heparin anticoagulation was adjusted to maintain an activated partial thrombin time of 1.5 times normal.

The baseline characteristics of the patients before VV-ECMO are summarized in Table 1. When VV-ECMO was started, the lung compliances of all three patients were very low (<20 mL/cmH_2_O). Thus, we applied an ultra-protective ventilation to prevent ventilator-induced lung injury (VILI). For ultra-protective ventilation, MV was adjusted to a peak inspiratory pressure ≤20 cmH_2_O and positive end expiratory pressure (PEEP) to 5–10 cmH_2_O. Tracheostomy was performed when lung compliance recovered to ~40 mL/cmH_2_O. Lung recruitment maneuver was applied when total lung atelectasis was seen at chest radiography. VV-ECMO weaning was considered when lung compliance had fully recovered (>40 mL/cmH_2_O) and percutaneous oxygen saturation was maintained at >95% while maintaining ultra-protective ventilation. Decannulation was performed when the patient tolerated a sweep gas flow rate of 0 L/min for 6–8 h.

Dexamethasone was administered from the beginning of VV-ECMO at 20 mg for 5 days, and at 10 mg daily for the next 5 days. Methylprednisolone was used per the Meduri protocol [7] when VV-ECMO weaning could not be considered after 10 days of dexamethasone administration.

Pharmacological management protocols, VV-ECMO support, and outcomes of the three patients are shown in Table 2.

### 2.1. Case 1

**Figure 1:**
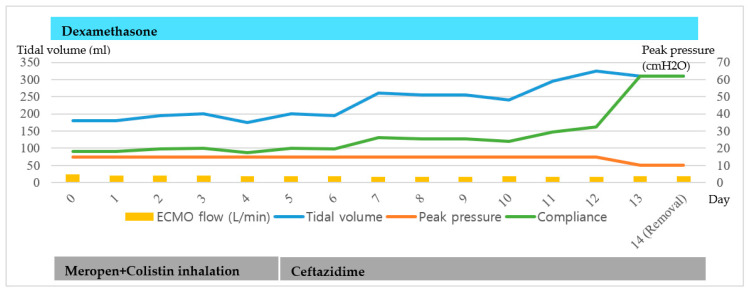
Clinical course of the patient 1 maintaining the Venovenous-Extracorporeal membrane oxygenation (VV-ECMO).

**Figure 2:**
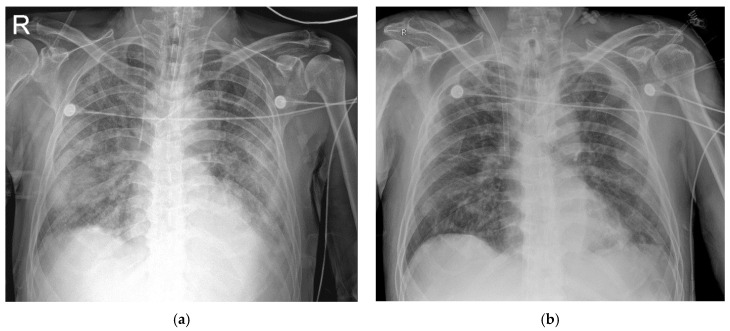
Chest radiography of the patient 1. (**a**) on the day of ECMO application (**b**) on the day of ECMO discontinuation.

A 69-year-old man diagnosed with COVID-19 was hospitalized for uncontrolled fever of a duration of one week on 27 March 2020. Right lower lung field infiltration was observed by chest radiography. He was treated with lopinavir/ritonavir, hydroxychloroquine, and ceftazidime and received convalescent plasma therapy. Because of respiratory distress and gas exchange deterioration, MV was applied on hospital day 9, and VV-ECMO on hospital day 13 because of increased oxygen requirements. Antibiotics were changed to colistin, meropenem, and dexamethasone, and were administered while VV-ECMO was maintained. After 11 days of VV-ECMO (hospital day 24), chest radiography findings and lung compliance showed signs of recovery. VV-ECMO was discontinued after 14 days (Figure 1 and Figure 2)

### 2.2. Case 2

**Figure 3:**
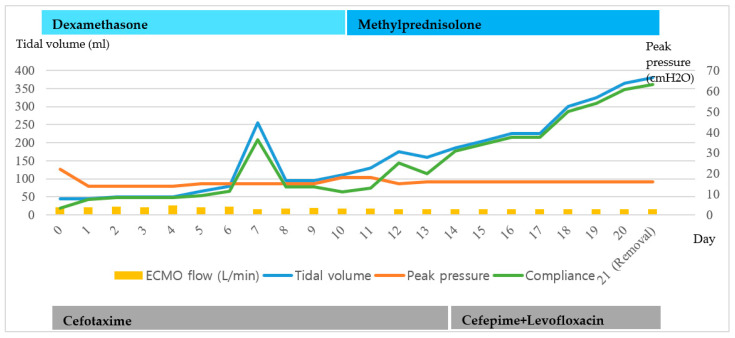
Clinical course of the patient 2 maintaining the VV-ECMO.

**Figure 4:**
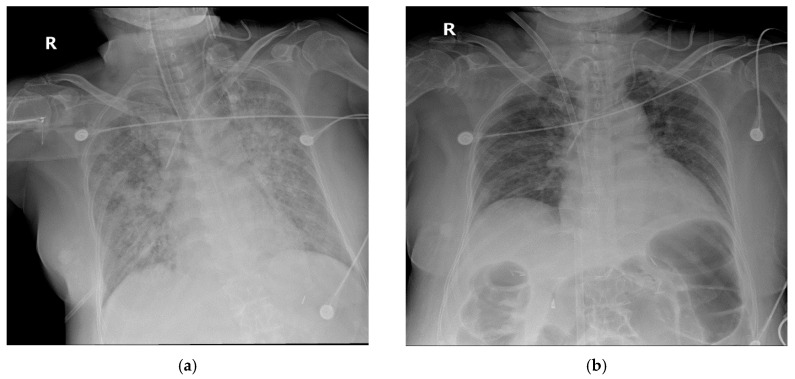
Chest radiography of the patient 2. (**a**) on the day of ECMO application (**b**) on the day of ECMO discontinuation.

Despite being hospitalized at another hospital for a week, a 56-year-old woman diagnosed with COVID-19 was transferred to our hospital on 7 June 2020, because of worsening respiratory distress. MV was applied just before being transfer. On arrival, extensive bilateral infiltration was observed by chest radiography. MV was applied at maximum settings and a neuromuscular block was administered. However, percutaneous oxygen saturation remained at approximately 50%. VV-ECMO was applied immediately (hospital day 1) and cefotaxime, convalescent plasma therapy, and dexamethasone were administered. On hospital day 18, chest radiography findings and lung compliance began to improve. VV-ECMO was discontinued on hospital day 21 (Figure 3 and Figure 4).

The pupillary light reflex was checked three times per day while the ECMO was maintained to avoid missing neurologic complications such as brain hemorrhage. On day 7, the response to light reflex was reduced from before, and all sedative drugs and the neuromuscular blocker were aborted to check the patient’s mental status. As a result, spontaneous respiration was recovered, and transpulmonary pressure was increased. After we checked the patient’s mental status, we administrated sedative drugs and neuromuscular blockers again.

### 2.3. Case 3

**Figure 5:**
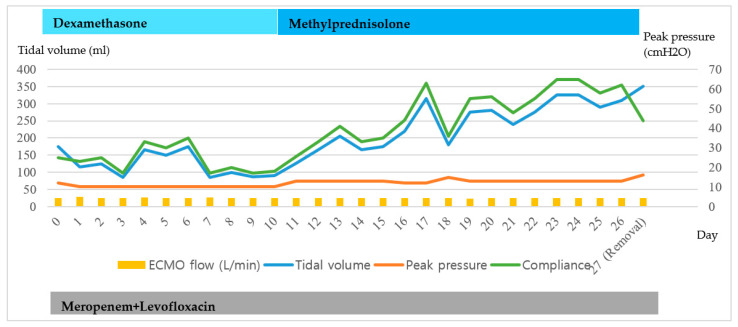
Clinical course of the patient 3 maintaining the VV-ECMO.

**Figure 6:**
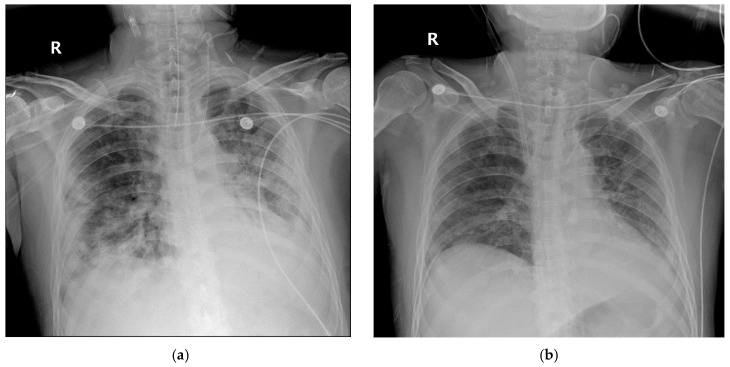
Chest radiography of the patient 3. (**a**) on the day of ECMO application (**b**) on the day of ECMO discontinuation.

A 58-year-old man diagnosed with COVID-19 10 days previously was transferred to our hospital on 8 June 2020 due to increased oxygen requirements. When he arrived, the high flow nasal cannula previously applied was set at fraction of inspired oxygen (FiO_2_) 1.0 and flow 60 L/min. Six days later (hospital day 6), MV was applied, because of respiratory distress, hypoxemia, and hypercapnia. Nevertheless, hypoxemic and hypercapnic respiratory failure worsened. VV-ECMO was implemented on hospital day 11. During VV-ECMO, meropenem, levofloxacin, convalescent plasma therapy, and dexamethasone were administered. On hospital day 33, chest radiography and lung compliance began to improve. VV-ECMO was discontinued on hospital day 38 (Figure 5 and Figure 6).

## 3. Discussion

Although our three patients had markedly lower lung compliance and poorer general conditions than have been reported in the majority of previous studies, all three successfully recovered from COVID-19-induced ARDS on VV-ECMO and an ultra-protective ventilation [8,9,10].

In patients with ARDS, MV may exacerbate or result in lung injury (called VILI) caused by biotrauma and volutrauma. Biotrauma causes pulmonary and systemic inflammatory response due to leukocyte activation and proinflammatory mediator release and may result in multi-organ failure. On the other hand, volutrauma induced by high lung volumes and distension of alveoli causes physical stress in lungs [11,12].

VILI decreases lung compliance by causing lung fibrosis, and lung compliance is an important predictor of ARDS outcome. Mean pre-ECMO lung compliance of survivors was 31 mL/cmH_2_O, while that of non-survivors was 20.7 mL/cmH_2_O [13]. Pre-ECMO lung compliances of our three patients were similar to those of non-survivors in this previous study (<20 mL/cmH_2_O), and thus, poor outcomes were expected.

Appropriate minute ventilation cannot be maintained by MV alone in most cases when ultra-protective ventilation is applied. However, when VV-ECMO is initiated, ultra-protective ventilation can be maintained and reduces VILI as compared with lung-protective ventilation [14]. Thus, we applied and maintained ultra-protective ventilation by applying peak inspiratory pressures of ≤20 cmH_2_O and PEEPs of ≤10 cmH_2_O to prevent VILI. VV-ECMO was maintained until lung compliance recovered to >40 mL/cmH_2_O, and subsequently, patients were weaned off VV-ECMO. Because an ultra-protective ventilation with lower tidal volume promotes atelectasis, we have applied lung recruitment maneuver whenever total lung atelectasis occurred.

When dexamethasone is administered to patients with moderate-to-severe ARDS, diffuse lung damage is reduced due to its anti-inflammatory and anti-fibrotic properties. Thus, dexamethasone may reduce mortality in moderate-to-severe ARDS and in mechanically ventilated COVID-19-induced ARDS patients [15,16]. We administered dexamethasone and methylprednisolone to reduce lung fibrosis and consider that this helped patient recovery.

Recently, convalescent plasma (CP) therapy has been used to boost the abilities of patients with severe COVID-19 to fight the virus [17]. Our patients also received CP therapy before VV-ECMO implementation or while on VV-ECMO. However, the effect of CP therapy in our patients is not clear. In Case 1, after CP infusion, oxygen requirements and fever improved for 3 days, but then the patient’s condition deteriorated and oxygen requirements increased, and thus, VV-ECMO was implemented.

## 4. Conclusions

Our experiences indicate that maintaining an ultra-protective ventilation with VV-ECMO aids recovery from COVID-19-induced ARDS. In addition, we suggest corticosteroids be considered to reduce lung fibrosis and injury.

## Figures and Tables

**Table 1:** Baseline characteristics of the three patients.

	Patient 1 (4/9)	Patient 2 (6/7)	Patient 3 (6/18)
Age (years)	69	56	58
Gender	Male	Female	Male
BMI	23.2	22.9	26.3
Hypertension	Yes	No	No
Diabetes mellitus	Yes	No	No
Bilateral lung involvement	Yes	Yes	Yes
SOFA score	9	7	10
APACHE II score	25	27	33
RESP score	1/Class III	5/Class II	3/Class II
Arterial blood gas analysis			
pH	7.4	7.3	7.3
PaO_2_ (cmH_2_O)	55.4	53.5	58.8
PaCO_2_ (cmH_2_O)	52.8	32.7	57.2
SaO_2_ (%)	89.2	52.5	86.2
Mechanical ventilation			
Tidal volume (mL)	180	45	175
PEEP (cmH_2_O)	5	14	14
PaO_2_/FiO_2_	55.4	32.7	58.8
Driving pressure (cmH_2_O)	10	8	12
Laboratory			
White blood cells (×10^9^/L)	9.1	16	11.3
Neutrophils (%)	83.4	90	88.5
Lymphocytes (%)	10.5	2	6
Platelet (×10^9^/L)	289	198	494
Creatinine (mg/dL)	0.5	0.5	0.5
Prothrombin time (%)	74	94	92
Activated partial thrombin time (s)	32.4	27.7	46.6
C-reactive protein (mg/dL)	22.6	13.9	22.3
Procalcitonin (ng/mL)	0.3	0.4	0.3
RASS score	−4	−4	−5

Abbreviations: BMI: Body mass index; SOFA: Sequential Organ Failure Assessment; APACHE: Acute Physiology and Chronic Health Evaluation; RESP: Respiratory ECMO Survival Prediction; PaO_2_: Arterial oxygen partial pressure; PaCO_2_: Arterial carbon dioxide partial pressure; SaO_2_: Arterial oxygen saturation; PEEP: Positive End Expiratory Pressure; FiO_2_: Fraction of inspired oxygen; RASS: Richmond Agitation-Sedation Scale.

**Table 2:** Pharmacological management, venovenous extracorporeal membrane oxygenation (VV-ECMO) support, and patient outcomes.

	Patient 1	Patient 2	Patient 3
Neuromuscular blockers	Yes	Yes	Yes
Steroids	Yes	Yes	Yes
	Hemodynamic support		
ECMO configuration	Veno-venous	Veno-venous	Veno-venous
Inflow line site	Right femoral vein	Right femoral vein	Right femoral vein
Inflow line size (Fr)	21	21	21
Reperfusion line site	Right IVJ	Right IVJ	Right IVJ
Reperfusion line size (Fr)	17	17	17
Oxygenator	PLS	EBS	PLS
	Medical therapy		
Antiviral	Lopinavir + ritonavir/Hydroxychloroquine	No	Rilpivirine/Hydroxychloroquine
Antibiotics	Meropenem + Colistin inhalation	Cefotaxime + Levofloxacin	Meropenem + Levofloxacin
Convalescent plasma therapy	Yes	Yes	Yes
	Outcomes		
Ventilation duration (days)	31	24	33
ECMO duration (days)	14	21	28
Bleeding *	No	No	No
Superinfection **	No	No	No
Tracheostomy	Yes	Yes	Yes
Survival on ECMO	Yes	Yes	Yes
Weaned from ECMO	Yes	Yes	Yes
ICU LOS (days)	37	26	6/14-
ICU survival	Yes	Yes	Yes
Hospital LOS (days)	65	61	64
Hospital survival	Yes	Yes	Yes

Abbreviations: ECMO: Extracorporeal membrane oxygenation; IVJ: Internal jugular vein; ICU: Intensive care unit; LOS: Length of stay. * ‘Bleeding’ defined as a case where a procedure is needed for hemostasis in brain, gastrointestinal track, intrathoracic, the site of a previous invasive procedure, and ECMO cannula site. ** ‘Superinfection’ defined as a new infection occurring in a patient with COVID-19 pneumonia.
